# Proprioceptive Cervicogenic Dizziness Care Trajectories in Patient Subpopulations: A Scoping Review

**DOI:** 10.3390/jcm12051884

**Published:** 2023-02-27

**Authors:** Joseph Gill-Lussier, Issam Saliba, Dorothy Barthélemy

**Affiliations:** 1 School of Rehabilitation, Faculty of Medicine, University of Montreal, Montreal, QC H3N 1X7, Canada; 2 Center for Interdisciplinary Research in Rehabilitation of Greater Montreal (IURDPM), CRIR, CIUSSS South-Center, Montreal, QC H3S 1M9, Canada; 3 Collège d’Études Ostéopathique de Montréal (CEOM), Montréal, QC H3G 1W7, Canada; 4 Division of Otolaryngology, Head and Neck Surgery—Otology and Neurotology, Montreal University Hospital Center (CHUM), University of Montreal, Montreal, QC H2X 3E4, Canada

**Keywords:** cervicogenic, cervical, proprioceptive, vertigo, dizziness, PCGD, whiplash

## Abstract

Proprioceptive cervicogenic dizziness (PCGD) is the most prevalent subcategory of cervicogenic dizziness. There is considerable confusion regarding this clinical syndrome’s differential diagnosis, evaluation, and treatment strategy. Our objectives were to conduct a systematic search to map out characteristics of the literature and of potential subpopulations of PCGD, and to classify accordingly the knowledge contained in the literature regarding interventions, outcomes and diagnosis. A Joanna Briggs Institute methodology-informed scoping review of the French, English, Spanish, Portuguese and Italian literature from January 2000 to June 2021 was undertaken on PsycInfo, Medline (Ovid), Embase (Ovid), All EBM Reviews (Ovid), CINAHL (Ebsco), Web of Science and Scopus databases. All pertinent randomized control trials, case studies, literature reviews, meta-analyses, and observational studies were retrieved. Evidence-charting methods were executed by two independent researchers at each stage of the scoping review. The search yielded 156 articles. Based on the potential etiology of the clinical syndrome, the analysis identified four main subpopulations of PCGD: chronic cervicalgia, traumatic, degenerative cervical disease, and occupational. The three most commonly occurring differential diagnosis categories are central causes, benign paroxysmal positional vertigo and otologic pathologies. The four most cited measures of change were the dizziness handicap inventory, visual analog scale for neck pain, cervical range of motion, and posturography. Across subpopulations, exercise therapy and manual therapy are the most commonly encountered interventions in the literature. PCGD patients have heterogeneous etiologies which can impact their care trajectory. Adapted care trajectories should be used for the different subpopulations by optimizing differential diagnosis, treatment, and evaluation of outcomes.

## 1. Introduction

### 1.1. Background and Rationale

The prevalence of dizziness among people of working age (18 to 65 years old) is 20%–30% [1,2,3,4], and it is the number one reason for medical consultations for people over 75 years old [5]. Cervicogenic dizziness, cervical vertigo, and cervicogenic vertigo are interchangeable terms that refer to dizziness that is closely associated with neck pain, neck injury, or neck pathology. Many consider it to be one of the most common causes of dizziness, as it contributes to major social costs, insurance claims and handicap [3,6,7,8]. Throughout this manuscript, dizziness is understood as a non-rotatory illusion of movement, accompanied by disequilibrium and lightheadedness.

As a consequence of the absence of a gold standard testing procedure, cervicogenic dizziness’s diagnosis is based on clinical presentation and the exclusion of other possible causes of dizziness [9,10,11]. However, researchers and clinicians should not only distinguish this syndrome from other pathologies, but should also distinguish between the many potential etiologies that can lead to cervicogenic dizziness [11]. Indeed, patients with cervicogenic dizziness are not a homogeneous group, and have been classified into subgroups of individuals who share similar clinical characteristics. They were categorized to account for the differences in clinical presentation and care trajectory, as well as the notion that subgrouping improves disease knowledge acquisition [11]. The care trajectory refers to the itinerary of a patient through the healthcare system and among the different actors over a continuous period from the onset of the illness to its resolution [12]. However, even if the concept of subgroups has developed over the past two decades, it is still poorly understood, underused in the clinical and research setting, and has not yet been systematically examined. Before undertaking this review, a preliminary search using Medline (Ovid), Embase (Ovid), All EBM Reviews (Ovid), and CINAHL (Ebsco) for existing scoping reviews and systematic reviews on the subject was conducted on 5 May 2021. Most reviews were narrative and, although very informative, related to the general concept of cervicogenic dizziness, implying different subsets of patients and lacking systematic reporting of charting methods. The few systematic reviews were not scoping reviews. Therefore, this scoping review focuses on the care trajectory of the most common subset of cervicogenic dizziness in an articulated scope of inquiry, that of proprioceptive cervicogenic dizziness (PCGD) [9,11,13,14,15].

PCGD is experienced as non-rotatory vertigo, instability and disequilibrium associated with neck pain caused by abnormal afferent cervical proprioceptive activity [3,9,16]. It corresponds to what numerous authors would refer to as cervicogenic dizziness. The term PCGD will be used in this article, as proposed by Devaraja (2018), because it is more precise and eliminates other possible causes of cervicogenic dizziness, such as cervical vascular etiology [11,17]. Thus, this review will focus exclusively on the potential proprioceptive etiology of cervicogenic dizziness. PCGD is a diagnosis of exclusion [3,9,10,11,18] and exhibits a complex and heterogeneous nature. Different groups of patients are diagnosed with PCGD [11]. The specific proprioceptive mechanisms leading to PCGD may be different across individuals [9,11] and there is still confusion regarding this clinical syndrome. Indeed, encounters with dizzy patients should be distinguishing into vascular, vestibular, central, metabolic, pharmaceutical, orthopedic, iatrogenic, psychological, optometric and somatosensory pathologies [9,10,11]. Comorbidities are often encountered in this complex clinical context. Accordingly, there is inappropriate and insufficient diagnostic accuracy and treatment related to PCGD, which often results in lengthy care trajectories [8]. Differential diagnoses, diagnostic criteria, optimal treatment [4], and outcome measures must be mapped out to help shorten care trajectories for these complex patients.

### 1.2. Objectives and Review Questions

Hence, the objectives of this scoping review are to clarify the conceptual boundaries of PCGD and to map out the main research designs used to study PCGD and the key characteristics of affected patient populations. To do so, we will: (1) systematically identify the key characteristics of the literature and populations that have PCGD and (2) classify accordingly the knowledge contained in the literature in regard to interventions, outcomes and diagnosis.

Therefore, our review question can be summarized by the following: How has PCGD been studied, diagnosed, evaluated and treated in the pertinent literature, considering the key characteristics of patient subpopulations? This question implies the following interrogations: (1) What are the main research designs used to study PCGD? (2) Which subpopulations of patients does a PCGD diagnosis represent? (3) Which common differential diagnoses are associated with those subpopulations? (4) What evaluation tools are mentioned to identify the diagnosis? (5) What interventions have been considered by researchers for the management of PCGD? (6) What outcome measures have been used?

## 2. Materials and Methods

Protocol and registration: This scoping review was informed by the Joanna Briggs Institute methodology [19]. As such, Preferred Reporting Items for Systematic Reviews and meta-analyses (PRISMA ScR) guidelines were followed to redact this systematic scoping review [20,21]. There was no a priori protocol published, because it was not recommended at the time of the beginning of the study.

Eligibility criteria: French, English, Spanish, Portuguese, and Italian articles were included in the review, as these languages are the ones fully understood by the reviewers. Randomized control trials, case studies, literature reviews, meta-analyses, and observational studies were included in this article. Expert advice, commentaries and letters were excluded to focus on higher evidence-level articles. Articles on animals were excluded because they cannot answer the research question. Research protocols were excluded as they do not yet contain a sample of patients and therefore cannot help to reach the scoping review’s objectives. Conference abstracts were excluded as they can potentially contain mistakes and have not been properly peer-reviewed.

Types of participants: Articles concerning patients that have proprioceptive cervicogenic dizziness (PCGD) with or without associated conditions were included to identify all subpopulations and pertinent information on the specific characteristics and care trajectories associated with PCGD. Articles related exclusively to patients that did not present PCGD, with dizziness of vascular, central, vestibular or pharmacological causes, were excluded as they do not contain information about PCGD. Studies on healthy subjects were included if they were related to PCGD.

Concept: The relevant care trajectory elements to extract included differential diagnoses, diagnostic and predictive tools, and evaluative assessments to measure change and interventions.

Context: Articles written before 2000 were excluded. The recent introduction of new diagnosis entities with similar clinical presentations such as persistent postural-perceptual dizziness (PPPD) and vestibular migraine suggests that only the recent literature informed by those new diagnoses can have homogeneous samples of PCGD. Additionally, the separation of cervicogenic dizziness from proprioceptive etiologies (PCGD) and cervicogenic dizziness from other etiologies (i.e., vascular) was not suggested before 2000, to our knowledge.

Information sources and search strategy: A first limited search of MEDLINE (Ovid) and CINAHL to analyze the text words contained in the title and abstract of retrieved papers, and of the index terms used to describe the articles, was performed. The PsycINFO, Medline (Ovid), Embase (Ovid), All EBM Reviews (Ovid), CINAHL (Ebsco), Web of Science and Scopus databases were then searched using a Boolean strategy recommended by the university’s research librarian. As an example, «((cervicogenic or cervical or proprioceptive) adj3 (vertigo* or dizziness)).ab,kf,ti.» was used to search Embase (Ovid). The rest of the strategies and corresponding databases can be consulted in Supplementary Appendix SA: SEARCH STRATEGY. The results yielded from this second step were exported in «.ris» format to the Covidence digital application to complete the review’s methodology on 14 June 2021.

Evidence screening and selection: Both the title and abstract screening and the full-text review were carried out by two independent reviewers (including the main author) to identify potential literature and exclude irrelevant articles. Conflicts were settled by the main author and another independent researcher in consensus. Two separate reviewers extracted information from the articles, and consensus was reached with the main author of this scoping review. Additionally, cross-referencing was used to access primary sources concerning themes such as measuring tools, competing diagnoses, and epidemiology. A particular effort was made to find grey literature through contact with the main authors on the subject, but no unpublished literature was recruited with this approach.

Extraction and data charting process: A pilot testing of the extraction tool available in Covidence software was conducted by separate reviewers. This resulted in the modification and personalization of the final extraction tool, which allowed for all relevant results to be extracted to meet the scoping review’s objective (Supplementary Appendix SB).

Data items: Due to feasibility considerations, we limited the amount of data that we reported to study designs, subpopulations, differential diagnoses, diagnostic tools, interventions and outcome measures.

Synthesis of result: A descriptive quantitative synthesis of the evidence is provided by a tabulation and census of articles that relate each aspect of the care trajectory. Review articles are treated separately in some figures, and are not considered in other figures in order to give a true representation of the literature and to avoid double counting of data. A descriptive narrative of evidence is also presented.

## 3. Results

### 3.1. Extracting and Charting the Results

**Figure 1:**
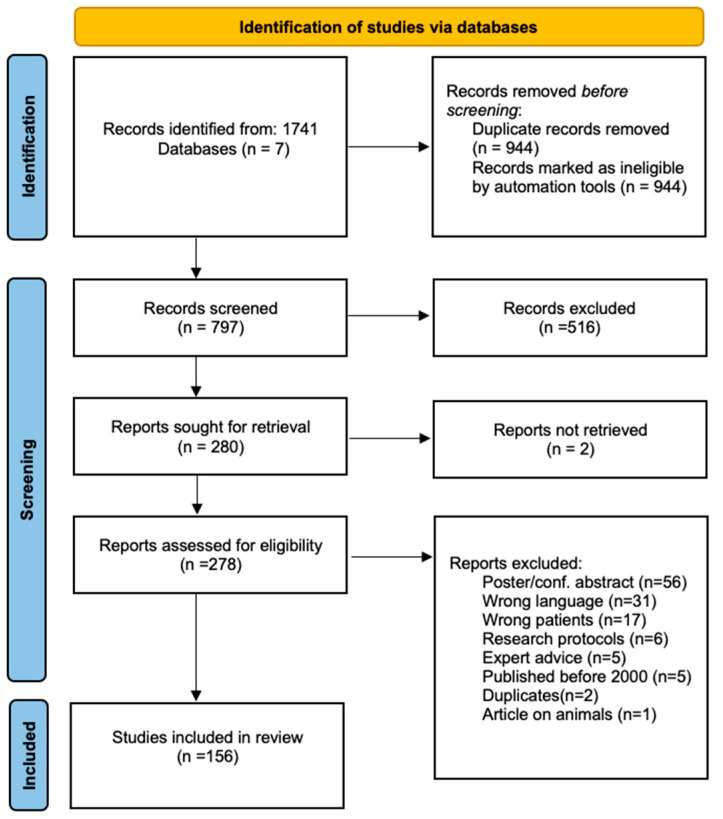
Prisma flow chart showing the identification of studies via databases.

The aforementioned methodology yielded 1741 studies. A total of 797 articles were left after Covidence automatically removed articles recognized to be duplicates (n = 944). The selected articles were then screened by two independent reviewers based on title and abstract. Some 516 studies were excluded because they were found irrelevant based on inclusion/exclusion criteria. A total of 281 articles were assessed for eligibility in a full-text selection process by two independent reviewers. Some 125 studies were excluded following a full-text review based on the exclusion criteria (see Supplementary Appendix SC for the list and reason for exclusion). Conflicts were settled by consensus both in the title and abstract selection stage, and the full-text selection stage with the input of a third party (the last author of this paper). Finally, 156 studies were identified and selected for inclusion in the scoping review. A detailed search decision flowchart is presented in Figure 1. 

#### 3.1.1. Study Designs

**Figure 2:**
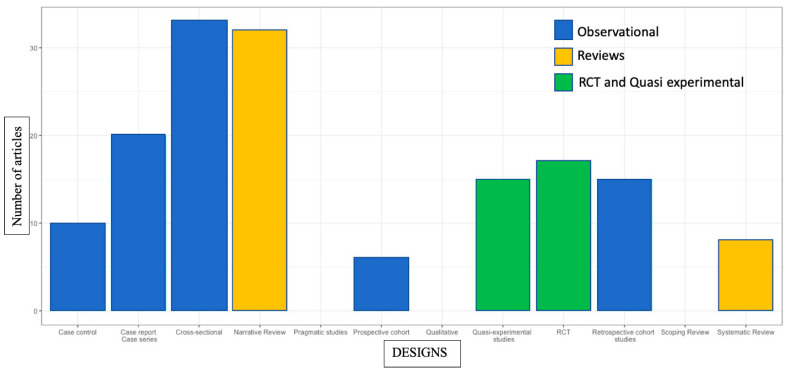
Research designs on proprioceptive cervicogenic dizziness. The number of articles selected by this scoping review is classified by study designs. RCT: randomized control trials.

A variety of different study designs were included: 17 randomized control trials (RCT) [22,23,24,25,26,27,28,29,30,31,32,33,34,35,36,37,38], 14 quasi-experimental studies [39,40,41,42,43,44,45,46,47,48,49,50,51,52], 84 observational studies of various designs (6 prospective cohort studies [53,54,55,56,57,58], 15 retrospective cohort studies [6,59,60,61,62,63,64,65,66,67,68,69,70,71,72], 20 case reports and case series [73,74,75,76,77,78,79,80,81,82,83,84,85,86,87,88,89,90,91,92], 33 cross-sectional studies [14,16,93,94,95,96,97,98,99,100,101,102,103,104,105,106,107,108,109,110,111,112,113,114,115,116,117,118,119,120,121,122,123], 10 case–control studies [15,124,125,126,127,128,129,130,131,132]), 9 systematic reviews [18,133,134,135,136,137,138,139,140], and 32 narrative reviews [1,3,4,7,9,10,11,141,142,143,144,145,146,147,148,149,150,151,152,153,154,155,156,157,158,159,160,161,162,163,164,165]. No qualitative studies, scoping reviews or pragmatic control trials have been published on PCGD. Figure 2 illustrates a quantitative synthesis of the study designs in the PCGD literature.

#### 3.1.2. Subpopulations of PCGD

**Figure 3:**
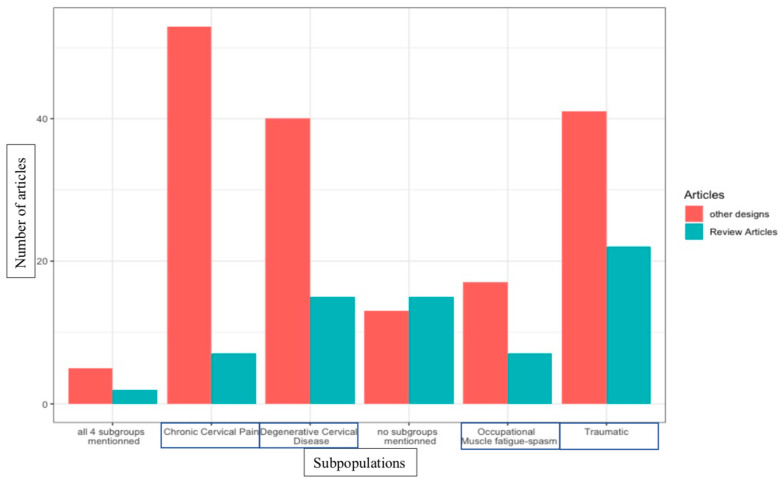
Quantitative synthesis of PCGD subpopulations’ occurrence in the literature. Articles mentioning more than one subpopulation were counted in each pertinent category, except when all four subgroups were mentioned. No subgroups: no subpopulations are specified in the articles.

A total of 81.9% of articles, reviews and other designs acknowledge at least one subpopulation in PCGD. Some 43.9% of articles acknowledge more than one subpopulation. A total of 28 of the 156 selected articles do not mention subpopulations in PCGD (18.1%). In total, four subpopulations of PCGD are identified in the literature: (A) chronic cervicalgia [7,13,14,15,22,24,25,26,28,29,30,31,34,36,37,41,45,46,50,56,58,60,61,63,64,65,66,68,73,74,75,77,78,81,82,84,85,86,89,90,92,95,98,99,100,109,110,115,116,118,120,124,126,142,146,148,153,163,166,167], (B) traumatic [1,6,7,9,10,11,13,16,23,24,29,31,37,38,39,42,45,50,55,62,63,64,65,66,69,75,77,81,82,86,87,88,90,95,96,98,99,103,106,107,109,112,114,116,119,124,125,127,137,138,140,141,142,143,145,148,149,150,152,154,157,158,162,163,164,168], (C) degenerative cervical disease [1,3,6,7,9,10,11,15,16,23,24,27,31,36,45,51,52,56,58,69,71,72,78,79,80,90,94,96,98,99,101,104,106,108,115,117,120,124,126,127,128,131,132,137,143,145,149,152,153,154,156,158,163,166,169,170], and (D) occupational postures and muscle fatigue or spasm [4,7,9,55,61,79,84,102,104,113,114,115,124,143,146,153,171] (see Table 1). Those potential etiological factors may alter in their specific ways the function of mechanoreceptors found in the smooth tissues (muscle, cartilage, tendons and ligaments) of the cervical region [4,16,146]. An illustrated quantitative synthesis of PCGD subpopulations’ occurrence in the literature may be found in Figure 3.

#### 3.1.3. Assessments Used for the Diagnosis of PCGD

- Differential diagnoses and their assessment tools

**Figure 4:**
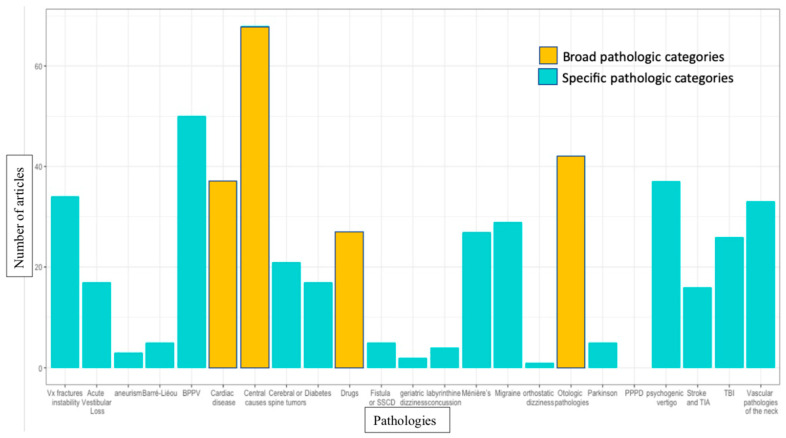
Pertinent differential diagnosis. SSCD: superior semicircular canal dehiscence syndrome; BPPV: benign paroxysmal positional vertigo; PPPD: persistent postural-perceptual dizziness; TIA: transient ischemic attack TBI: traumatic brain injury Vx: vertebral.

As PCGD is a diagnosis of exclusion, the literature mentions 23 potential pathologies or group of pathologies to be ruled out. These are presented in a quantitative illustrated synthesis in Figure 4. Central causes [15,16,23,26,27,29,30,31,33,34,35,40,41,42,44,46,50,52,54,57,58,59,63,65,66,67,68,70,71,73,74,76,82,83,85,89,93,95,96,98,101,102,103,104,106,107,108,109,110,112,114,115,118,120,123,125,126,128,130,131,132,166], benign paroxysmal positional vertigo (BPPV) [15,16,22,23,24,25,26,29,30,31,32,39,42,54,55,56,57,59,63,65,66,67,70,74,76,82,83,84,86,88,90,99,101,102,103,104,107,108,109,110,111,112,114,115,125,126,132,156,166] and otologic pathologies [22,23,24,28,31,32,33,34,40,44,49,53,54,56,58,59,60,61,68,74,75,76,77,78,80,81,82,83,92,95,100,102,104,106,107,108,112,123,125,128,131] are the three most commonly occurring differential diagnosis categories in the literature on PCGD. The occurrence of differential diagnoses in articles mentioning different subpopulations of PCGD is presented in Table 2.

**Figure 5:**
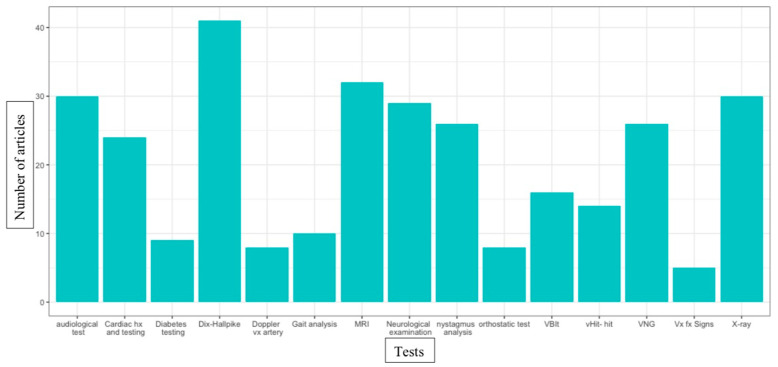
Pertinent tests for differential diagnosis. Cardiac hx: cardiac history, vx: vertebral, MRI: magnetic resonance imaging, VBIt: vertebrobasilar insufficiency testing, vHIT-hit: video head impulse test-head impulse test, VNG: videonystagmography, Vx Fx signs: vertebral fracture signs, X-ray: cervical spine X-ray imagery.

Furthermore, a total of 32 measuring tools contributing to the differential diagnosis process were mentioned in the literature. The most mentioned tools enabling this differential diagnosis process in the literature are presented in a quantitative illustrated synthesis in Figure 5. The Dix-Hallpike maneuver [10,15,16,22,23,24,26,29,30,31,35,38,39,42,55,56,57,59,65,66,75,76,81,82,84,86,90,99,101,104,107,108,109,112,115,123,125,128,130,132], magnetic resonance imaging (MRI) [6,31,42,56,58,60,67,70,71,78,81,82,86,90,92,93,95,98,99,100,106,107,108,109,110,114,115,117,126,128,132,166], cervical spine x-ray imagery (X-ray) [15,27,28,35,39,46,56,57,58,69,70,71,77,81,84,89,90,93,96,99,100,102,106,116,119,123,124,128,129] and audiological testing [15,41,42,47,51,55,56,58,60,62,67,69,70,74,81,82,93,95,98,99,101,106,107,108,109,110,114,123,125,129] are the four most reported tools to guide the differential diagnosis process. The Dix-Hallpike maneuver can identify BPPV, MRI can objectify some central causes, cervical x-rays can identify a vertebral fracture and audiological testing helps in the diagnosis of different otologic pathologies.

- Inclusive diagnostic tools (rule-in)

**Figure 6:**
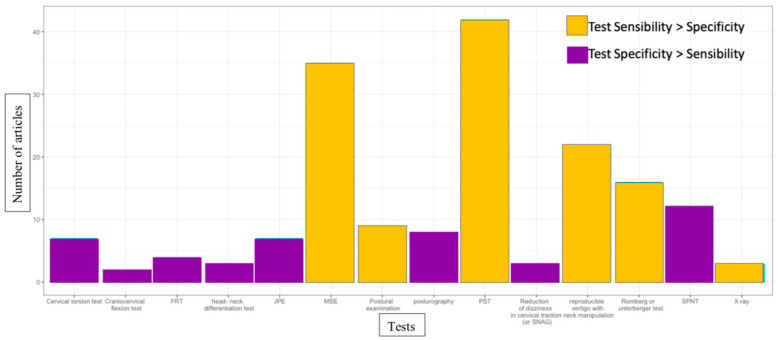
Clinical tests (to «rule-in») PCGD. FRT: flexion rotation test, JPE: joint position error test, MSE: manual spinal evaluation, PST: palpation for segmental tenderness, SNAG: sustained natural apophyseal glide SPNT: smooth pursuit neck torsion test.

While exclusion diagnosis implies clinicians will «rule out» other pathologies, some clinical tests can also help to inform clinicians by trying to «rule in» PCGD. A list of the most often-cited tests is found in Figure 6. The two most cited clinical tests mentioned in the literature are palpation for segmental tenderness [6,10,16,23,24,25,26,28,29,34,35,36,40,43,45,51,55,60,61,66,73,74,76,77,78,81,84,85,87,90,93,95,98,100,103,104,107,109,120,126,128,129] and manual spinal evaluation [10,16,22,23,26,27,28,31,35,36,40,41,51,55,56,59,61,69,72,73,74,75,77,78,81,82,85,87,90,93,103,104,125,126,128].

#### 3.1.4. Interventions and Outcome Measures

**Figure 7:**
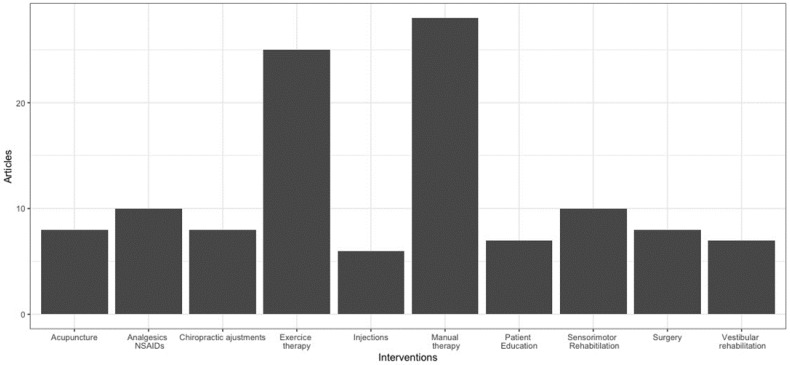
Interventions on PCGD in the literature.

Many therapeutic interventions were found (n = 34) in the PCGD literature (see Table 3). These included modalities from physiotherapy, Chinese medicine, pharmacology, allopathic medicine, chiropractic medicine, and other approaches. Across subpopulations, exercise therapy [22,23,25,26,27,31,34,37,38,40,42,51,59,60,65,74,81,82,84,85,87,90,125,128,166] and manual therapy [22,23,24,25,26,27,28,31,33,36,38,40,41,42,46,59,60,76,78,79,82,84,87,90,101,107,124,128] are the most commonly encountered intervention in the relevant literature, as shown in Figure 7. The occurrence of interventions in articles mentioning different subpopulations of PCGD are presented in Table 4.

**Figure 8:**
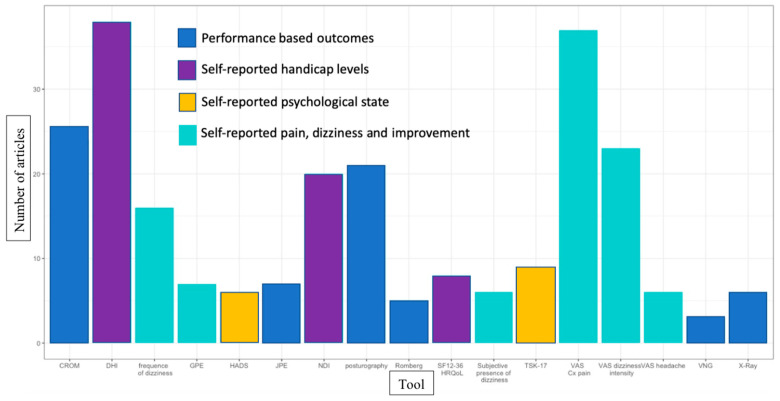
Tools to measure change CROM: cervical range of motion, DHI: dizziness handicap inventory, GPE: global perceived effect, VNG: videonystagmography, HADS: Hospital Anxiety and Depression Scale, JPE: joint position error test, NDI: neck disability index, HRQoL: health-related quality of life, TSK: Tampa Scale for Kinesiophobia, VAS: visual analog scale.

**Figure 9:**
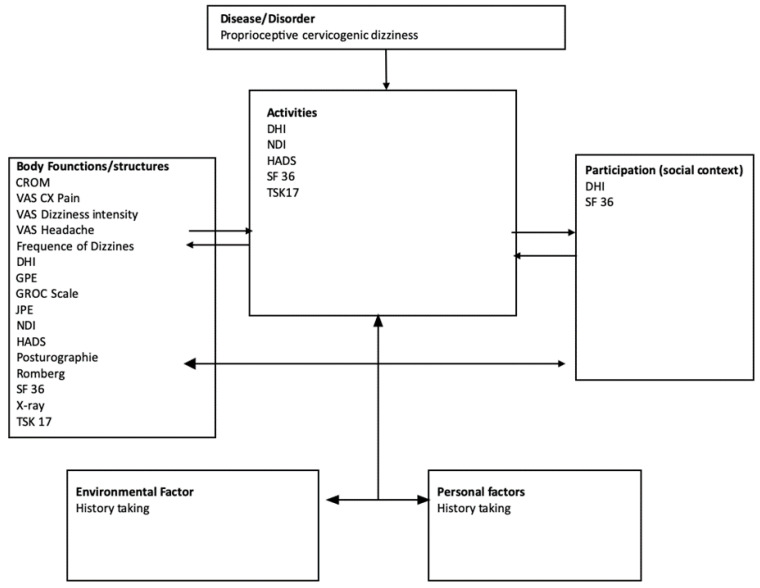
Measurement instruments in proprioceptive cervicogenic dizziness: an ICF classification (some measurement instruments are suitable in more than one ICF component). CROM: cervical range of motion, DHI: dizziness handicap inventory, GPE: global perceived effect, GROC-scale: global rating of change, HADS: Hospital Anxiety and Depression Scale, JPE: joint position error test, NDI: neck disability index, HRQoL: health-related quality of life, TSK: Tampa Scale for Kinesiophobia, VAS: visual analog scale.

While evaluating the efficiency of treatment, the 17 most commonly encountered outcome measures relevant to PCGD literature are presented in Figure 8. A total of 77 measuring tools were found in the literature. The four most cited measures of change were: dizziness handicap inventory (DHI) [16,23,24,25,26,27,28,29,30,31,32,33,34,35,42,56,66,68,76,77,78,81,85,89,90,95,98,104,107,108,109,110,117,120,128,130], Visual analog scale (VAS) for neck pain [16,23,24,25,26,27,30,31,34,40,41,51,54,60,64,68,71,73,74,76,77,78,81,84,86,90,100,106,109,110,116,117,118,126,128,166], cervical range of motion (CROM) [22,23,24,25,26,28,29,33,40,41,46,51,52,60,76,78,84,85,87,106,108,109,120,128] and posturography [16,23,24,25,26,29,33,34,39,40,43,46,47,48,62,85,95,106,107,110,119] (see Figure 8). Across subpopulations, measures of change used in trials are fairly similar (see Table 5). Dizziness is a multidimensional rehabilitation problem [172]. These dimensions should be considered when measuring treatment effectiveness. Figure 9 uses the International Classification of Functioning, Disability and Health to classify the most common health outcomes of functioning and disability found in the PCGD literature. The body function category (Figure 9) regroups many self-reported and performance-reported outcomes related to the many dimensions of PCGD: proprioceptive and sensorimotor performance (posturography, joint position error test (JPE) and Romberg), self-reported pain (VAS for cervicalgia and headaches), the amplitude of cervical movement (CROM), frequency and intensity of dizziness, level of self-perceived disability (DHI, neck disability index (NDI)), quality of life (SF-36), medical imagery of the cervical spine (X-Ray), and levels of anxiety and depression (Hospital Anxiety and Depression Scale (HADS)). Some self-reported outcomes measuring body functions also comprise items relative to activity and, to a much lesser degree, to the participation category (DHI and SF-36). Indeed, dizziness can limit social participation and engagement and the ability to work, and may even exclude a patient from his profession [172]. DHI and SF-36 do not include specific items related to the social contribution of patients, such as their ability to work, their days on sick leave and the personal economic impact of the disease. In the literature, only one article reported on sick leave in PCGD [105].

## 4. Discussion

This article focused on the following central questions: (1) What are the main research designs used to study PCGD? (2) Which subpopulations of patients does a PCGD diagnosis represent? (3) What common differential diagnoses are associated with those subpopulations? (4) What evaluation tools are mentioned to identify the diagnosis? (5) What interventions have been considered by researchers for management? (6) Which outcome measures have been used?

To our knowledge, this is the first scoping review undertaken on the topic of PCGD. Four subpopulations of PCGD have been identified: chronic neck pain, degenerative cervical disease, traumatic and occupational subpopulations (muscle spasm). Central causes of dizziness and BPPV are the most often-mentioned potential diagnoses that compete with PCGD. The Dix-Hallpike maneuver is the most cited tool to inform differential diagnosis. Manual therapy and exercise therapy are the most studied interventions in the field. DHI is the most often-encountered measure of change used in this literature.

### 4.1. Designs

Many study designs were selected for this review (see Figure 2). Randomized control trials represent only approximately 10% of the selected literature. Observational studies are the most common designs. No qualitative protocols on the subject were found in any database; this type of research should be encouraged because evidence-informed practice, value-based healthcare approaches and patient participatory paradigms require a more qualitative knowledge of the experience of patients suffering from PCGD in order to focus on what matters to them [173].

### 4.2. Subpopulations

Four subpopulations of PCGD have been identified that reflect the relatively heterogeneous population of PCGD. The reasons that some people develop PCGD and others do not, even though they are part of traumatic, degenerative cervical disease, muscle spasms or chronic neck pain populations, remain unknown [158]. Maybe differences in sensorial strategies between individuals could account for that. Patients keener on using proprioceptive input will be more at risk of developing PCGD in comparison with patients who rely more heavily on vestibular or visual cues for posture and gait. As patients can be part of more than one subpopulation of PCGD, research could investigate how cumulating etiological factors could predict poor prognosis [174]. Because PCGD pertains to different subpopulations, it is a «cross-cutting complaint» that concerns different specialties. As such, our results support the Bárány Society’s recommendation to form multidisciplinary research teams to study PCGD [175], and their calls for interdisciplinary efforts in the clinic.

### 4.3. Competing Diagnoses, Differential Diagnosis and Comorbidity

Since PCGD is an exclusion diagnosis, central causes, cardiac disease and otological pathologies are among the four most cited pathological categories to be ruled out (see Figure 4). Unfortunately, these categories lack precision because they regroup numerous pathologies and are too elusive to effectively orient the differential diagnosis process and inform clinicians. BPPV, on the other hand, is the most cited specific diagnosis in this literature. It is also cited often as an important diagnosis to rule out, no matter what the subpopulation of PCGD is (see Table 2). It is therefore no surprise that the relatively simple Dix-Hallpike maneuver is the most cited test to rule out competing pathologies with PCGD (see Figure 5). Indeed, this test associated with adequate nystagmus analysis; paroxysmal presentation of symptoms and history taking can signal a BPPV diagnosis, but only for posterior canal issues. Lateral canal issues are not objectified with this test.

Knowledge of subpopulations could orient clinicians toward the most accurate and pertinent use of resources in terms of diagnostic tools. Indeed, certain differential diagnostic processes are more often encountered in articles recognizing specific subpopulations of PCGD.

In the literature on the traumatic subpopulation of PCGD, vertebral fractures and particularly traumatic brain injury are more often mentioned than in any other subpopulation. Additionally, there are relatively fewer mentions of the necessity to exclude cardiac diseases in the literature on the traumatic subpopulation of PCGD compared with other subpopulations. Clinicians could orient their diagnostic process toward a rather orthopedic direction rather than a cardiovascular direction in this subpopulation. While MRI, neurological examination and X-ray are cited in the literature on PCGD to help with differential diagnoses (see Figure 5), there are relatively few mentions of orthopedic examination for vertebral fracture signs and ligament testing. This could indicate that clinicians rely more on imagery than clinical testing, and could use clinical testing more, especially with the traumatic population.

In the literature on the degenerative cervical disease subpopulation of PCGD, cardiac disease and drug-induced dizziness are relatively more often cited than in any other subpopulation. This might be because the degenerative cervical disease subpopulation is more likely to be elderly, have cardiac conditions and be exposed to multiple drug issues [176]. Indeed, cardiac history and testing is the 7th most cited evaluation used in differential diagnosis (see Figure 5).

Incidentally, psychogenic vertigo is cited relatively more often in both the traumatic and the degenerative cervical disease subpopulations than in the other subgroups. This could be explained by the potential psychological impacts related to trauma or ageing. Paradoxically, no mention of psychological assessment is present in the tests to inform differential diagnosis. More psychological testing should be carried out in a neurotological context, as vertigo and dizziness can also cause anxiety, panic and depression, and these could in turn also cause dizziness [177].

Other important aspects to discuss are persistent postural perceptual dizziness (PPPD) and vestibular migraine. PPPD was recognized by the International Classification of Diseases (ICD-11) only in 2017 [178], and vestibular migraine has been described by the members of the Bárány Society only since 2012 [179]. Although migraine is cited relatively often as a diagnosis of exclusion in PCGD, especially in the traumatic and chronic cervicalgia subpopulations, there is no golden standard to «rule in» migraine and diagnosis is based on clinical presentation [180]. This result supports the importance of controlling for migraine and developing subgroup analysis for migraine as a confounding factor in future interventional studies, as prompted by the Bárány Society’s recent milestone article on ‘Cervical Dizziness’ [175]. Persistent postural perceptual dizziness is a common long-lasting cause of dizziness [178]. Paradoxically, it is not mentioned in the exclusion process of PCGD. Migraine and persistent postural perceptual dizziness are both exclusion diagnoses and can co-exist with other conditions. This situation adds to diagnosis uncertainty.

Early diagnosis and rehabilitation could optimize health outcomes for patients and add value to healthcare by reducing the social-economic burden of disease. One of the main issues with the lengthy care trajectory of PCGD is that despite being an exclusion diagnosis, it may also coexist with other disorders, and often does. Moreover, in elderly people at risk of falls, road accident victims suffering from post-concussion syndrome or whiplash, and patients suffering from neck pain or chronic headaches, 45.2% to 84% of patients have potentially one or more diagnoses in addition to PCGD [11,123,181]. In these subpopulations, dizziness is associated with higher levels of disability and more psychosocial consequences compared to patients in the same groups without dizziness [13,113,135,154,182]. This multi-morbid situation makes the trajectory of care longer, and often results in therapeutic wandering for these patients, and a greater social and economic burden. There is a lack of a single gold standard test or accepted clinical prediction rule to limit diagnosis uncertainty [18]. Only one article has studied the possibility of combining different tests to shorten the exclusion process [16]. The issue of multi-morbidity calls for investigation of clinical prediction rules and the specificity of tests to «rule-in» PCGD. Indeed, while sensitive tests such as manual spinal evaluation and palpation for segmental tenderness are very often used in the literature, potentially more specific tests [10] such as cervical torsion [10,16,64,84,90,97,115], the head–neck differentiation test [10,97,123], joint position error test [10,16,50,65,87,90,98] and smooth pursuit neck torsion test [10,16,23,26,50,57,64,65,99,103,125,128] have a relatively lower rate of occurrence in the literature. Unfortunately, in comparison with the literature reporting tools to «rule out» other pathologies, the literature reporting clinical testing that is useful to «rule in» PCGD with more specific tools is scarce, and therefore should be encouraged (see Figure 5 and Figure 6).

### 4.4. Measuring Change

In PCGD, many outcome measures are needed not only because of its multidimensional nature, but because self-reported outcomes and perceived level of handicap poorly correlate with the measurement of the level of sensorimotor performance [183]. This suggests they rely on other constructs [183]. The most commonly encountered outcome measures in PCGD are DHI, VAS for cervical pain, CROM and posturography (see Figure 8). Posturography and JPE are the only tests that can be found both among the tools for inclusion and for measuring change (see Figure 6 and Figure 8), and this raises the question of their potential combined specificity and sensitivity to change. These six tools should be used to facilitate comparison between trials and meta-analysis of outcomes, and a psychological outcome such as HADS should also be included. Social engagement and personal economic impacts of disease should be reported in PCGD. As PCGD remains elusive in its exact aetiopathogenesis, primary clinical outcomes and secondary «mechanistically based» outcomes should also help to establish a basis for hypothesized pathophysiological mechanisms [175].

### 4.5. Interventions

Manual therapy and exercise therapy are generally the most common interventions encountered in the literature. However, surgeries were considered more often for degenerative cervical disease subpopulations suffering from PCGD than in any other subpopulation (see Table 4). The reason for this might be that surgeries are aimed at degenerative changes and herniated disc issues rather than for dizziness itself, even if they may have an indirect impact on dizziness. In the same way, injections were mostly reported in the literature for the chronic cervical pain subpopulation, as they are a common treatment for chronic neck pain. Patient education was the third most studied intervention in the muscle spasm (occupational) subpopulation. Indeed, patient education about occupational habits aims to reducing muscle spasms, and indirectly could impact dizziness.

Knowledge of subpopulations’ characteristics should also be reflected in the multimorbid context of PCGD. It enables the clinician to consider not only the type of intervention but the strategy of intervention that might be considered.

### 4.6. Limitations

Relevant sources of information may have been omitted in the literature written in languages that were not included in the review, notably Chinese and German articles. Another limitation of this study is that the proprioceptive etiology of cervicogenic dizziness is yet to be recognized by The International Classification of Vestibular Disorders. It is a working definition and is the most plausible cause of dizziness in cervicogenic dizziness, but still requires further investigation into its pathophysiological mechanism. In this scoping review, as no quality assessment of protocols was performed, the validity of the literature has not been put to the test. Care should be taken while interpreting the results.

In the differential diagnosis process, since some authors simply excluded general cardiac, central and otologic conditions without naming any particular pathology, specific conditions pertaining to those categories might be under-represented in our results in Figure 4. Additionally, relatively recent diagnoses in otology and neurotology make some pathologies unlikely to have been put forward in the differential diagnosis of PCGD before 2010, and even today. For example, a potentially relevant competing diagnosis such as PPPD might have been reported using other terms such as phobic postural vertigo or visual vertigo, but only two articles mentioned each pathology.

Some 43.9% of articles acknowledge more than one subpopulation. This introduces bias in subpopulation analysis. Particularly in the chronic cervicalgia analysis, 34 of the 53 articles also recognize at least one other subpopulation for PCGD, and in so doing, introduce bias to our data. For example, these articles could mention vertebral fractures or instability as a pathology to exclude, because the authors recognize the potential contribution of trauma or degenerative cervical disease subpopulations in PCGD.

## 5. Conclusions

This is the first scoping review of the literature on PCGD, to the authors’ knowledge. Qualitative methods are inexistent in the literature on PCGD. The specific characteristics of PCGD patients differ according to their etiological categories. Subpopulation knowledge should inform subgroup analysis in PCGD trials and observational studies as well as clinical practice. Namely, there are four main subpopulations of PCGD: chronic cervical pain, traumatic, degenerative cervical disease and occupational. These subgroups have different care trajectories according to commonly encountered pathologies, probable comorbidities, usual red flags, and treatment strategies. This raised awareness will have important impact on future research in relation to subgroup analysis and in clinical practice, as it enables optimized differential diagnosis, treatment, and evaluation. Studies should also investigate the reason that some patients from a single subpopulation develop PCGD and others do not; more randomized control studies are needed. Trials should use common outcome measures encompassing all dimensions of PCGD, including the social and economic categories, to facilitate future systematic reviews and elucidate pathophysiological mechanisms. Future studies should report on clinical testing to «rule in» PCGD. 

## Figures and Tables

**Table 1:** Subpopulations, clinical presentation and hypothesized etiological mechanism.

Subpopulations	Clinical Presentation	Hypothesized Etiological Mechanism
**Chronic cervical pain**	Patients with cervical pain for more than 12 weeks with no history of trauma or presence of muscle spasm that present dizziness.	Pain potentially alters proprioception
**Traumatic**	Patients have a history of ^1^ WAD or ^2^ PCS. Along with dizziness and cervical pain, patients may present the following symptoms: ataxia, unsteadiness of gait, postural imbalance, limited neck range of motion and potentially headache.	Pain, limitation of movement, and strains of joint capsules, paravertebral ligaments, and cervical musculature can alter cervical proprioception
**Degenerative cervical disease**	Mostly elderly populations presenting dizziness associated with degenerative cervical changes and cervical pain. Some patients may complain of headaches, or shoulder pain and some radicular symptoms or possible.	Histological changes and inflammatory processes can alter cervical proprioception
**Occupational muscle spasm or fatigue**	Sedentary populations that present dizziness associated with neck muscle fatigue or spasm without trauma. Patients could present limited cervical range of motion.	Muscle spasm may alter proprioceptive input

^1^ WAD: whiplash associated disorders. ^2^ PCS: post-concussion syndrome.

**Table 2:** Occurrences of differential diagnoses in articles mentioning different subpopulations of PCGD.

Traumatic	Degenerative Cervical Disease	Chronic Cervicalgia	Occupational (Muscle Spasm)
^1^ BPPV (n = 22)	Central causes (n = 22)	Central causes (n = 28)	^1^ BPPV (n = 11)
Central causes (n = 19)	Cardiac disease (n = 17)	^1^ BPPV (n = 23)	Central causes (n = 9)
^3^ Vx fracture instability (n = 17)	^1^ BPPV (n = 16)	Cardiac disease (n = 20)	Cardiac disease (n = 8)
Migraine (n = 13)	^3^ Vx fracture instability (n = 14)	^3^ Vx fracture instability (n = 19)	Vascular pathologies of the neck (n = 7)
Psychogenic vertigo (n = 15)	Psychogenic vertigo (n = 13)	Migraine (n = 19)	Migraine (n = 7)
^2^ TBI (n = 12)	Drugs and vascular pathologies of the neck (n = 11 for both)	Otologic pathologies (n = 18)	Drugs (n = 6)

^1^ BPPV: Benign paroxysmal positional vertigo ^2^ TBI: traumatic brain injury ^3^ Vx: vertebral.

**Table 3:** Intervention modalities in Proprioceptive cervicogenic dizziness *.

Chinese Medicine	Physiotherapy	Pharmacology	Surgery-Injection
Acupuncture Tuina Acupressure Herbs	Manual therapy Transcutaneous electrical nerve stimulation Vestibular rehabilitation Dry needling Exercise therapy Sensorimotor rehabitilation Ultrasound Termal therapy Sustained natural apophyseal glide	Non-steroidal anti-inflammatory drugs Acetaminophen Analgesics Betahistine Muscle relaxant	Total disc replacement (TDR) Medial branch blocks (MMBs) Occipital nerve blocks (GON) Trigger point injections (TPI) Mepivacaine, bupivacaine Anterior cerical discetomy and fusion (ACDF) Percutaneous laser disc decompression (PLDD) and disc decompression. Botulinum toxin injection Coblation discoplasty Carbon fiber fusion cage (CIFC)

* Other modalities included are chiropractic adjustments; chuna manual therapy; helical patches; cervical traction; and patient education.

**Table 4:** Occurrences of interventions in articles mentioning different subpopulations of proprioceptive cervicogenic dizziness.

Traumatic	Degenerative Cervical Disease	Chronic Cervicalgia	Occupational (Muscle Spasm)
Exercise therapy (n = 10)	Manual therapy (n = 9)	Manual therapy (n = 15)	Manual therapy (n = 4)
Manual therapy (n = 10)	Surgery (n = 8)	Exercise therapy (n = 13)	Exercise therapy (n = 3)
Sensorimotor rehabilitation (n = 6)	Analgesic NSAID ^1^ (n = 6)	Chiropractic adjustments (n = 7)	Patient education (n = 3)
Analgesic NSAID ^1^ (n = 5)	Exercise therapy (n = 6)	Injection (n = 5)Analgesic NSAID ^1^ (n = 5)	Analgesic NSAID ^1^ (n = 3)
Patient education (n = 5)	Acupuncture (n = 4)	Acupuncture (n = 5)	Chiropractic adjustments (n = 2)

^1^ NSAID: Non-steroidal anti-inflammatory drugs.

**Table 5:** Measures of change used across subpopulations.

Traumatic	Degenerative cervical disease	Chronic cervicalgia	Occupational (muscle spasm)
^1^ DHI (n = 15)	^1^ DHI (n = 16)	^2^ VAS Cervical pain (n = 24)	^1^ DHI (n = 5)
^2^ VAS Cervical pain (n = 12)	^2^ VAS Cervical pain (n = 16)	^1^ DHI (n = 23)	^2^ VAS Cervical pain (n = 5)
Posturography (n = 10)	^4^ CROM (n = 9)	^4^ CROM (n = 14)	^5^ TSK-17 (n = 3)
^3^ NDI (n = 9)	^3^ NDI (n = 8)	^3^ NDI (n = 13)	^3^ NDI (n = 2)
^4^ CROM (n = 7)	^2^ VAS intensity and frequency of dizziness both (n = 8)	^2^ VAS intensity and frequency (n = 13)	Posturography (n = 2) and ^4^ CROM (n = 2)

^1^ DHI: dizziness handicap inventory; ^2^ VAS: visual analog scale; ^3^ NDI: neck disability index; ^4^ CROM: cervical range of motion; ^5^ TSK: Tampa Scale for Kinesiophobia.

## Data Availability

Data is contained within the article and in the Supplementary Materials.

## Supplementary Materials

The following supporting information can be downloaded at: https://www.mdpi.com/article/10.3390/jcm12051884/s1, Appendix SA: Search strategy. Supplementary Appendix SB: Data extraction instrument. Supplementary Appendix SC: Sources excluded following full-text review.
