# Supplementary material for: Proprioceptive Cervicogenic Dizziness Care Trajectories in Patient Subpopulations: A Scoping Review
Source: J Clin Med. 2023 Feb 27;12(5):1884. doi: 10.3390/jcm12051884 (PMC10003866; doi:10.3390/jcm12051884)
Supplement: Supplementary file 1 [file jcm-12-01884-s001.zip › jcm-2207799-supplementary.pdf]

# Supplementary Appendix SA: SEARCH STRATEGY

| *Database* | *strategy* |
|---|---|
| *PsycInfo Ovid 16* | *((cervicogenic or cervical or proprioceptive) adj3 (vertigo* or dizziness)).ab,id,ti.* |
| *Embase Ovid 393* | *((cervicogenic or cervical or proprioceptive) adj3 (vertigo* or dizziness)).ab,kw,ti.* |
| *Medline Ovid 302* | *((cervicogenic or cervical or proprioceptive) adj3 (vertigo* or dizziness)).ab,kf,ti.* |
| *All EBM Reviews Ovid 131* | *((cervicogenic or cervical or proprioceptive) adj3 (vertigo* or dizziness)).mp.* |
| *CINAHL Ebsco 137* | *TI ( (cervicogenic or cervical or proprioceptive) N3 (vertigo* or dizziness) ) OR AB ( (cervicogenic or cervical or proprioceptive) N3 (vertigo* or dizziness) ) OR SU ( (cervicogenic or cervical or proprioceptive) N3 (vertigo* or dizziness) )* |
| *Web of Science 284* | *Topic=(cervicogenic or cervical or proprioceptive) NEAR/3 (vertigo* or dizziness)* |
| *Scopus* | *TITLE-ABS-KEY ( ( cervicogenic or cervical or proprioceptive ) W/3 ( vertigo* or dizziness ) )* |

# Supplementary Appendix SB: DATA EXTRACTION INSTRUMENT

### Section 1 - General information

**Study ID**
Doi, PMID, etc.

**Title**

**Authors**

**Lead author contact details**
E-mail

**Institution**

**Country in which the study conducted**
- United States
- UK
- Canada
- Australia
- Italy
- France
- Other

**Data collection**
If not experimental study, RCT or controlled study, then just clic «other» and explain.
- Single center
- Multi center
- Other

### Section 2 - Epidemiology, Etiology and Physiopathology

**Epidemiology**

**Social and economic impact - on the patient**

**Social and economic impact - on society**

**Prevalance**

**Incidence**

**Aetiology: state the mentionned aetiology or aetiologies of PCGD in article. (Chronic, induced pain or occupational,Degenerative cervical disease,Autonomous Nervous System ,Autonomous Nervous System,Associated headache/migraines,Trauma (WAD, PCS, orthopedic,etc), or other..)**

**Clinical presentation (symptoms)**

**Physiopathology**

**Other information (relevant)**

### Section 3 - Characteristics and Study Population

**Study design**

**Inclusion criteria**

**Exclusion criteria**

**Participants**

**N**

**Age**

**Sexe**

**Population type**
- Active
- Sedentary
- Other

**Comorbidity**

**Secondary gains**
If patients are covered by insurance or not. Here write anything related to malingering and potential secondary gains from insurance.

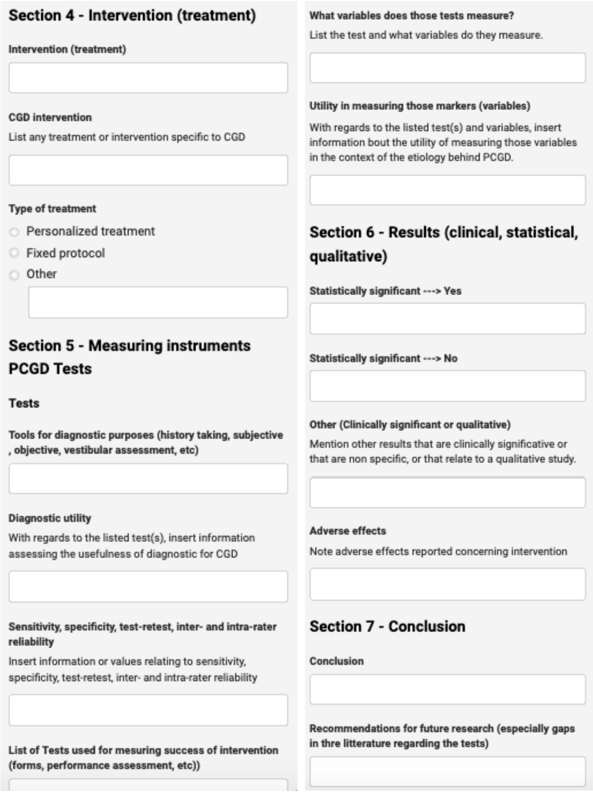

### Section 4 - Intervention (treatment)

**Intervention (treatment)**

**CGD intervention**

List any treatment or intervention specific to CGD

**Type of treatment**

- Personalized treatment
- Fixed protocol
- Other

### Section 5 - Measuring instruments PCGD Tests

**Tests**

**Tools for diagnostic purposes (history taking, subjective , objective, vestibular assessment, etc)**

**Diagnostic utility**

With regards to the listed test(s), insert information assessing the usefulness of diagnostic for CGD

**Sensitivity, specificity, test-retest, inter- and intra-rater reliability**

Insert information or values relating to sensitivity, specificity, test-retest, inter- and intra-rater reliability

**List of Tests used for mesuring success of intervention (forms, performance assessment, etc))**

**What variables does those tests measure?**

List the test and what variables do they measure.

**Utility in measuring those markers (variables)**

With regards to the listed test(s) and variables, insert information bout the utility of measuring those variables in the context of the etiology behind PCGD.

### Section 6 - Results (clinical, statistical, qualitative)

**Statistically significant ---> Yes**

**Statistically significant ---> No**

**Other (Clinically significant or qualitative)**

Mention other results that are clinically significative or that are non specific, or that relate to a qualitative study.

**Adverse effects**

Note adverse effects reported concerning intervention

### Section 7 - Conclusion

**Conclusion**

**Recommendations for future research (especially gaps in thre litterature regarding the tests)**

# Supplementary Appendix SC: SOURCES EXCLUDED FOLLOWING FULL-TEXT REVIEW

Abdel-Baki F, Koraym HK, Helal MH

Posturography in cervical vertigo

/ 2000;123(2):P235

2000 /

06/07/21 Poster/conference abstract

Bai, Xiao-Dong; Zhang, Shao-Feng; Xing, Geng-Yan

Correction of atlas drift by chiropractic treatment for the vertigo of the cervical syndrome

Chinese Journal of Clinical Rehabilitation / 2005;9(34):112-113

China Journal of Clinical Rehabilitative (P.O. Box 1200, Shenyang 110004, China) 2005

/

07/09/21 Poster/conference abstract

Bajaj, Poonam Ramesh; Hathiram, Bachi; Khattar, Vicky; Contractor, Aashish;

Chaturvedy, Gaurav; Punjabi, Raashi; Chhabria, Anjali

Physical therapy in non-vertiginous dizziness

Neurorehabilitation and Neural Repair / 2018;32(4-5):455

Netherlands SAGE Publications Inc. 2018 /

07/07/21 Poster/conference abstract

Becker-Bense, Sandra; Huppert, Doreen

Less common, but clinically important episodic vertigo syndromes

Fortschritte der Neurologie Psychiatrie / 2021;89(5):221-232

Germany Georg Thieme Verlag 2021 /

05/10/21 Other language then french, english, spanish, italian and portuguese

Brandt, T.; Huppert, D.

Cervical vertigo: Head motion-induced dizzy spells in acute neck pain

European Journal of Neurology / 2017;24(Supplement 1):735

Netherlands Blackwell Publishing Ltd 2017 /

07/07/21 Poster/conference abstract

Brown, J.J.

Cervical Contribution to Balance: Cervical Vertigo

The Head-Neck Sensory Motor System 2012;():

2012

09/10/21 published before 2000

Bulman, Shauna M.; Virani, Shane; Schneider, Kathryn J.; Harris, Ashley D.; Barlow, Karen M.; Yeates, Keith O.; Brooks, Brian L.

Dizziness in children and adolescents with a history of concussion: Symptoms and functional limitations

Clinical Journal of Sport Medicine / 2018;28(3):e73

Netherlands Lippincott Williams and Wilkins 2018 /

12/09/21 Poster/conference abstract

Bus, G.; Van Den Broeck, C.; Vijverman, V.; Malone, S.; Vaes, P.; Oostendorp, R.A.B.

The development and peer evaluation of a screening tool for cervicogenic dizziness

Manual Therapy / 2016;25((Vaes, Bus, Van Den Broeck, Vijverman, Malone) Manual Therapy Vrije Universiteit Brussel-Belgium, Brussels, Belgium(Vaes, Oostendorp) Allied Health Sciences, Radboud University, Nijmegen, Netherlands):e148

Netherlands Churchill Livingstone 2016 /

06/07/21 Poster/conference abstract

Cao Y

Clinical observations on acupuncture treatment of 56 cervical vertigo cases

/ 2002;21(06):8

2002 /

12/09/21 Poster/conference abstract

Chaibi, Aleksander; Tuchin, Peter J.

Chiropractic spinal manipulative treatment of migraine headache of 40-year duration using Gonstead method: a case study.

Journal of Chiropractic Medicine Sep 2011;10(3):189-193

New York, New York Elsevier B.V. 2011 Sep

13/07/21 Wrong patient population (dizziness of vascular, central, vestibular or pharmacological causes)

CHEN S-l, XU W, YAO L

Integrated traditional Chinese and western medicine on cervical vertigo

/ 2013;():

2013 /

12/09/21 Poster/conference abstract

Cossu, M.; Cigolini, M.; Veronese, A.

Static stabilometry in vertiginous disorder of cervical origin

Riabilitazione / 1997;30(2):49-59

Italy Masson SpA (Via F.lli Bressan 2, Milan 20126, Italy) 1997 /

Cossu, M.; Rega, V.

New diagnostic and therapeutic prospects in vertigo caused by cervical whiplash

Riabilitazione / 1997;30(3):105-119

Italy Masson SpA (Via F.lli Bressan 2, Milan 20126, Italy) 1997 /

09/09/21 published before 2000

Cui LJCombine Qingxuan Shujing

Decoction with abdominal acupuncture in treating cervical vertigo for 44 cases/

2015;13(5):45

2015 /

12/09/21 Poster/conference abstract

Dai Ning

Treatment of 175 Cervical Vertigo Cases by Acupuncture and Clinostatic Manual Traction

/ 2002;21(1):18

2002 /

12/09/21 Poster/conference abstract

Ding Xi-Rui, Gu Yong-Ming, Liu Su-Fen

Scalp acupuncture in combination with point-pressing in the treatment of 22 cases of cervical vertigo

/ 2001;21(3):57

2001 /

12/09/21 Poster/conference abstract

Deng ZK, Jiang YH

Qushi Tongluo therapy on the treatment of cervical vertigo for 70 cases

/ 2015;13(8):49

2015 /

12/09/21 Poster/conference abstract

Doijiri, R.; Kaijimoto, K.; Konaka, K.; Moriwaki, H.; Miyashita, K.; Nagatuka, K.; Uno, H.; Naritomi, H.

How commonly is stroke found in patients with isolated vertigo/dizziness attack?Cerebrovascular Diseases / 2010;29(SUPPL. 2):175

S. Karger AG 2010 /

16/07/21 Poster/conference abstract

Ellis, Michael J; McDonald, Patrick J; Olson, Ashley; Koenig, James; Russell, Kelly

Cervical spine dysfunction following pediatric sports-related head trauma.

The Journal of Head Trauma Rehabilitation / 2019;34(2):103-110

US Lippincott Williams & WilkinsUS 2019 /

09/10/21 Wrong patient population (dizziness of vascular, central, vestibular or pharmacological causes)

Erdem, H.R.; Duyur Çakit, B.; Ünlü, I.; Nacir, B.; Karagöz, A.; Saraçoğlu, M.

The evaluation of dizziness and balance in patients with cervical myofascial pain syndrome

Journal of Rheumatology and Medical Rehabilitation 2008;19(3):146-152

2008

22/11/21 unable to obtain

Erdogdu, Deniz Dulgeroglu; Karsli, Pinar Bora; Cengiz, Damla; Binici, Serpil; Cakci, Aytul; Ozdek, Ali; Donmez, Havva Keklikoglu

Clinical features of cervicogenic vertigo patients inconsistent with the diagnosis of central and vestibular vertigo

Turkiye Fiziksel Tip ve Rehabilitasyon Dergisi / 2013;59(SUPPL. 1):295

Turkish Society of Physical Medicine and Rehabilitation 2013 /

13/07/21 Poster/conference abstract

Euype ES; Marchetti GF

Case report of cervicogenic dizziness using an orthopaedic physical therapy approach.

Journal of Orthopaedic & Sports Physical Therapy ;39(1):A63-A63

Alexandria, Virginia JOSPT, Inc. d/b/a Movement Science Media

13/07/21 Poster/conference abstract

Fan Z, Huang GF, Huang W

Curative efficacy of electro acupuncture combined with herbal fomentation on cervical vertigo

/ 2015;31(11):37

2015 /

12/09/21 Poster/conference abstract

Fang J

Observation of curative effect on fixed spin reduction of spinal manipulation therapy for cervical vertigo

/ 2010;23(2):99

2010 /

12/09/21 Poster/conference abstract

Galli, J; Tartaglione, T; Calo, L; Ottaviani, F

Os odontoideum in a patient with cervical vertigo: a case report.

American journal of otolaryngology / 2001;22(5):371-3

United States 2001 /

02/08/21 Wrong patient population (dizziness of vascular, central, vestibular or pharmacological causes)

Gang, Q; Yong, Z

Subtle adjustment of the cervical spine combined with Shu Jing Ding Xuan Decoction for cervical vertigo

JOURNAL OF ACUPUNCTURE AND TUINA SCIENCE 2015;13(1):22-26

2015

09/10/21 Wrong patient population (dizziness of vascular, central, vestibular or pharmacological causes)

Gao ZG, Fu GB, Ke ZB

Treatment of cervical vertigo with manipulations combined with acupuncture

/ 2005;12(04):342005 /

09/09/21 Poster/conference abstract

Gong FM, Yang XB

Observations on the efficacy of stiletto needle acupuncture plus conventional acupuncture in treating cervical vertigo

/ 2015;34(6):553

2015 /

12/09/21 Poster/conference abstract

Gu C-L, Chen X-D, Zhu S-G, Lin M-C, Liu W-B, Dai J-Q

Effect of three vertigo-stopping needles on neurohumor of patients with cervical vertigo: a controlled trial

/ 2016;26(2):25

Elsevier B.V. 2016 /

17/07/21 Wrong patient population (dizziness of vascular, central, vestibular or pharmacological causes)

GU, Chun-lei; CHEN, Xiang-dong; ZHU, Si-gang; LIN, Ming-chun; LIU, Wen-bo; DAI, Jia-qing

Effect of three vertigo-stopping needles on neurohumor of patients with cervical vertigo: a controlled trial

World Journal of Acupuncture - Moxibustion / 2016;26(2):25-30

Netherlands Elsevier B.V. 2016 /

09/10/21 duplicate

Guo JH, Ma SZ, Liu YS

Clinical evaluation of diagnosis and treatment integration scheme in cervical vertigo

/ 2015;24(3):402

2015 /

12/09/21 Poster/conference abstract

Han, Jianlong; Zuo, Jinliang; Zhu, Dengsong; Gao, Chunzheng

The correlation between SNPs within the gene of adrenergic receptor and neuropeptide Y and risk of cervical vertigo.

Journal of clinical laboratory analysis / 2018;32(5):e22366

United States 2018 /

13/07/21 Wrong patient population (dizziness of vascular, central, vestibular or pharmacological causes)

Hahn, T.; Klessinger, S.

Response to cervical medial branch blocks in patients with cervicogenic vertigo

European Spine Journal / 2017;26(11):3036

Netherlands Springer Verlag 2017 /

21/07/21 Poster/conference abstract

Hauswirth J

Cervicogenic dizziness: diagnosis and manual therapeutic treatment.

Manuelle Therapie May 2008;12(2):80-93

Georg Thieme Verlag Stuttgart 2008 May

22/07/21 Other language then french, english, spanish, italian and Portuguese

Heitkamp, HSJ

Manual Therapy for Cervicogenic Dizziness: Long-Term Outcomes of a Randomised Trial

PHYSIOSCIENCE 2015;11(2):84-86

2015

21/07/21 Other language then french, english, spanish, italian and Portuguese

Holzl, M.; Hulse, M.

The efficiency of spinal manipulation in otorhinolaryngology. A retrospective long-term study

HNO / 2004;52(3):227-234

Germany Springer Verlag (Tiergartenstrasse 17, Heidelberg D-69121, Germany) 2004 /

17/07/21 Other language then french, english, spanish, italian and Portuguese

Holzl, M; Behrmann, R; Biesinger, E; von Heymann, W; Hulse, R; Arens, C

Selected otorhinolaryngological symptoms in functional disorders of the upper cervical spine and temporomandibular joints

HNO 2018;66(3):237-249

2018

21/07/21 Other language then french, english, spanish, italian and Portuguese

Hu, C.-N.; Wang, S.-H.

Clinical observation on oblique Ban-pulling tuina manipulation plus acupuncture for cervical vertigo

Journal of Acupuncture and Tuina Science 2016;14(1):26-30

2016

01/08/21 Wrong patient population (dizziness of vascular, central, vestibular or pharmacological causes)

A-Yong HUANG; Wei ZHOU; Hong-Gang ZHONG; Yong-Dong ZHANG; Qing-Sheng WU; Yu-Zhong SUN; Guo-Qiang LI.

Evaluation of diagnosing cervical vertigo with computerized static posturography

Zhongguo gu shang = China journal of orthopaedics and traumatology / 2008;21(5):345-348

China 2008 /

12/09/21 Other language then french, english, spanish, italian and portuguese

Huang, Fan; Zhao, Siyi; Dai, Lin; Feng, Zitong; Wu, Zhennan; Chen, Jia; Guo, Rusong; Tian, Qiang; Fan, Zhiyong; Wu, Shan

Tuina for cervical vertigo: A systematic review and meta-analysis of randomized controlled trials.

Complementary therapies in clinical practice / 2020;39(101225531):101115

England 2020 /

09/10/21 Wrong patient population (dizziness of vascular, central, vestibular or pharmacological causes)

Huo Qingping, Liang Fang, Wei Dezhi

Internal medical regimen for treatment of cervical vertigo by integrative medicine

/ 2013;():

2013 /

12/09/21 Poster/conference abstract

Jager S

Cervical vertigo in manual therapy.

Pt Zeitschrift fur Physiotherapeuten 08// 2004;56(8):1398-1412

Richard Pflaum Verlag GmbH & Co. KG 2004 08//

05/10/21 Other language then french, english, spanish, italian and portuguese

Jayaram, Prathap; Patel, Shounuck; Bowen, Jay

THE ROLE OF OZONE PROLOTHERAPY AS AN ADJUNCT THERAPY TO BOTULINUM TOXIN INJECTIONS IN THE TREATMENT OF CERVICAL DYSTONIA AND VERTIGO IN POST-CONCUSSION SYNDROME: A CASE REPORT.

American Journal of Physical Medicine & Rehabilitation Supplement 2014;():a30-1

Baltimore, Maryland Lippincott Williams & Wilkins 2014 Supplement

11/09/21 Poster/conference abstract

Kadanka, Zdenek; Bednarik, J.

Cervical vertigo - fiction or reality?

Ceska a Slovenska Neurologie a Neurochirurgie / 2018;81(5):521-527

Czechia Czech Medical Association J.E. Purkyne (E-mail: andrea.opletalova@meditorial.cz) 2018/

09/09/21 Other language then french, english, spanish, italian and portuguese

Kazmierczak, Henryk; Pawlak-Osinska, Katarzyna

Cervical vertigo - Ethiology, diagnosis and therapy

Polski Merkuriusz Lekarski / 2005;19(111):456-458

Poland Medpress (ul. Slonki 67, Warsaw 02-814, Poland) 2005 /

09/10/21 Other language then french, english, spanish, italian and portuguese

Khemraj, R.; Klein, A.

The use of cervical manipulation and neuromuscular re-education to decrease symptoms of cervicogenic dizziness: A case study

Journal of Manual and Manipulative Therapy / 2010;18(4):228-229

Maney Publishing 2010 /

16/07/21 Poster/conference abstract

King, H.H.

Manual therapy effects in patients with cervicogenic dizziness

Journal of the American Osteopathic Association 2014;114(10):810

2014

09/09/21 expert advice/commentaries/letters

Kitano, H.; Hakuba, A.; Hatada, K.; Yamane, H.

The diagnosis of dizziness, vertigo, ataxia and psychogenic unsteadiness and treatmenty by a neurologist

Equilibrium Research / 2001;60(1):7-15

Japan Japan Society for Equilibrium Research (39 Kawabata Higash Maruta-cho, Sakyo-ku, Kyoto 606, Japan) 2001 /

19/07/21 Other language then french, english, spanish, italian and portuguese

Konig, Stefan A; Goldammer, Axel; Vitzthum, Hans-Ekkehart

Anatomical data on the craniocervical junction and their correlation with degenerative changes in 30 cadaveric specimens.

Journal of neurosurgery. Spine / 2005;3(5):379-85

United States 2005 /

09/10/21 Wrong patient population (dizziness of vascular, central, vestibular or pharmacological causes)

Kristjansson, Eythor; Treleaven, Julia

Sensorimotor function and dizziness in neck pain: implications for assessment and management.

The Journal of orthopaedic and sports physical therapy / 2009;39(5):364-77

United States 2009 /

17/07/21 expert advice/commentaries/letters.

Krupka, B.; Krupka, J.; Schusterova, B.; Preisova, I.; Vlachova, I.; Herzig, R.

Somatosensory cervical vertigo and vertebrobasilar insufficiency - A differential diagnostic and

therapeutic problem

Rehabilitace a Fyzikalni Lekarstvi / 2002;9(3):80-85

Czech Republic Czech Medical Association J.E. Purkyne (Sokolska 31, P.O. Box 88, Praha 2 120 26, Czech Republic) 2002 /

26/11/21 unable to obtain

Landel, R.

Use of traction to treat cervicogenic dizziness: A case study

Physiotherapy (United Kingdom) / 2015;101(SUPPL. 1):eS818

Elsevier Ltd 2015 /

19/07/21 Poster/conference abstract

Lawrence, Dj

Mulligan sustained natural apophyseal glides and Maitland mobilisations for cervicogenic dizziness.

Focus on Alternative & Complementary Therapies 09// 2014;19(3):165-166

Malden, Massachusetts Wiley-Blackwell 2014 09//

19/07/21 expert advice/commentaries/letters.

Lehmann, N.; Moebus, S.; Michels, Thomas

Cervical vertigo - The combined application of local anaesthesia with supplementary acupuncture

Deutsche Zeitschrift fur Akupunktur / 2005;48(4):6-14

Germany Elsevier GmbH 2005 /

21/07/21 Other language then french, english, spanish, italian and portuguese

Li, Wei; Tan, Luo; Wang, Shu-qin

Sixty-five cases of cervical vertigo treated by needling cervical jiaj points in combination with acupoint pressing

World Journal of Acupuncture - Moxibustion / 2013;23(2):48-50

Netherlands Elsevier (P.O. Box 211, Amsterdam 1000 AE, Netherlands) 2013 /

09/09/21 Wrong patient population (dizziness of vascular, central, vestibular or pharmacological causes)

Li, Z.-Q.

Observation on the therapeutic efficacy of acupuncture plus tuina for cervical vertigo

Journal of Acupuncture and Tuina Science 2016;14(5):366-372

2016

27/07/21 Wrong patient population (dizziness of vascular, central, vestibular or pharmacological causes)

Li, Haiyan; Yu, Ting; Cheng, Pan; Qin, Siyu; Jiao, Lin; Chen, Rixin

Moxibustion for cervical vertigo: A protocol for a systematic review and meta-analysis.

Medicine / 2020;99(31):e21405

United States 2020 /

07/08/21 research protocols

Li Zhi

Clinical observation of 'dear-headed' acupuncture combined with massotherapy on cervical vertigo re-sulting from cervical spondylosis

/ 2004;26(2):124

2004 /

12/09/21 Poster/conference abstract

Liu JZ, Ai SC, Li JS

Blood-letting puncture combined with acupuncture cupping for treating cervicogenic vertigo: a study of 119 cases

/ 2015;35(6):14252015 /

12/09/21 Poster/conference abstract

Liu, Nong-Jun

Sequential rehabilitative treatment versus nonsequential combination therapy for cervical vertigo

Chinese Journal of Clinical Rehabilitation / 2005;9(18):24-25

China Journal of Clinical Rehabilitative (P.O. Box 1200, Shenyang 110004, China) 2005 /

07/09/21 Poster/conference abstract

Lundblad, Mark

SYMPTOM INSTABILITY AND A PROPOSED CLASSIFICATION SYSTEM FOR THE PHYSICAL THERAPY MANAGEMENT OF SPORTS-RELATED CONCUSSION.

International journal of sports physical therapy / 2019;14(5):835-844

United States 2019 /

09/10/21 expert advice/commentaries/letters.

Luth, Carl; Bartell, Desiree; Bish, Michaela; Yudd, Andrew; Palaima, Mary; Cleland, Joshua A.

The effectiveness of vestibular rehabilitation therapy vs conservative treatment on dizziness: a systematic review and meta-analysis.

Physical Therapy Reviews 10// 2019;24(5):229-238

Philadelphia, Pennsylvania Taylor & Francis Ltd 2019 10//

09/10/21 Wrong patient population (dizziness of vascular, central, vestibular or pharmacological causes)

Madhusudhan Rao, V.; Geetha Rani, K.S.; Manikandan, B.; Priyadardhini, G.

Dizziness in cervical spondylitis is due to recalibration of vestibular apparatus signals by cervical proprioceptive afferents

Biomedicine (India) / 2017;37(4):593

India Indian Association of Biomedical Scientists 2017 /

12/09/21 Poster/conference abstract

Martinez, M.; Hays, A.

Pulsed radiofrequency of the bilateral C2 nerve roots for the treatment of severe refractory upper cervical dysfunction induced vertigo

Regional Anesthesia and Pain Medicine / 2012;37(6):

Lippincott Williams and Wilkins 2012 /

12/09/21 Poster/conference abstract

Militskova, Alena; Mukhametova, Elvira; Baltina, Tatiana

Latent myogenic trigger points effects on upright stance

European Journal of Clinical Investigation / 2019;49(Supplement 1):196

Netherlands Blackwell Publishing Ltd 2019 /

19/07/21 Poster/conference abstract

Morrison, F

Research Compact Evaluation of Paraclinical Tests in the Diagnosis of Cervicogenic Dizziness

MANUELLE THERAPIE 2016;20(3):107-+

2016

21/07/21 Other language then french, english, spanish, italian and Portuguese

Ning D

Comparative Study on Clinical Therapeutic Effects of Acupoint-to-acupoint Needling and Medication for Cervical Vertigo

/ 2005;1():011 2005 /

27/07/21 Other language then french, english, spanish, italian and Portuguese

Oyelowo, Tolu

Rapid outcomes observed in a patient with cervicogenic headache following spinal manipulation therapy and massage.

Alternative medicine research yearbook 2013. / 2014;(Medical Treatment of Physical Illness [3363]Bronfort, G., Nilsson, N., Haas, M., Evans, R., Goldsmith, C. H., Assendelft, W. J., & Bouter, L. M. (2004). Non-invasive physical treatments for chronic/recurrent headache. Cochrane Database Syst Rev 2004;(3):C):325-328

Hauppauge, NY, US Nova Biomedical BooksUS 2014 /

18/10/21 Poster/conference abstract

Peng, Baogan; Pang, Xiaodong; Li, Duanming; Yang, Hong

Cervical spondylosis and hypertension: a clinical study of 2 cases.

Medicine / 2015;94(10):e618

United States 2015 /

09/10/21 Wrong patient population (dizziness of vascular, central, vestibular or pharmacological causes)

Poeck, K.

Medico-legal assessment of so-called whiplash injury

Aktuelle Neurologie / 2002;29(6):288-294

Germany Georg Thieme Verlag (Rudigerstrasse 14, Stuttgart D-70469, Germany) 2002 /

17/07/21 Other language then french, english, spanish, italian and Portuguese

Qin, Jingxiang; Liu, Wuzhong; Zhu, Jun; Weng, Wei; Xu, Jiaming; Ai, Zisheng

Health related quality of life and influencing factors among welders.

PloS one / 2014;9(7):e101982

United States 2014 /

18/07/21 Wrong patient population (dizziness of vascular, central, vestibular or pharmacological causes)

Refshauge, K.; Shirley, D.; Sawkins, K.

Dizziness related to dysfunction of the cervical spine: A systematic review of treatment

Physiotherapy (United Kingdom) / 2011;97(SUPPL. 1):eS1139

Elsevier Ltd 2011 /

18/07/21 Poster/conference abstract

Reid, S

Cervicogenic Dizziness - an Overview of Diagnosis and evidence-based Therapy

MANUELLE THERAPIE 2016;20(3):116-121

2016

21/07/21 Other language then french, english, spanish, italian and Portuguese

Reid, S.; Rivett, D.; Callister, R.; Katekar, M.

The identification of patients with cervicogenic dizziness

Physiotherapy (United Kingdom) / 2011;97(SUPPL. 1):eS1581

Elsevier Ltd 2011 /

15/07/21 Poster/conference abstract

Reid, S.; Callister, R.; Snodgrass, S.; Katekar, M.; Rivett, D.

Long-term outcomes of Mulligan sustained natural apophyseal glides and maitland passive joint mobilisations for chronic cervicogenic dizziness: A randomised trial

Physiotherapy (United Kingdom) / 2015;101(SUPPL. 1):eS1270-eS1271

Elsevier Ltd 2015 /

18/10/21 Poster/conference abstract

Beatriz Moral Saiz , Monica Grande Alonso, Ana Mınguez Zuazo, Roy La Touche, Sergio Lerma Lara

Balance disorders in patients with cervicogenic dizziness. A descriptive study.

Gait & Posture ;49():156-156

Elsevier B.V.

18/07/21 Poster/conference abstract

Sanders, Marc W.; Newell, David; Ramsoskar, Johan; Kristiansen, Kim D.; Pearse, Greg; Buvarp, Trym; Rix, George

A comparison of the effectiveness of manual therapy, exercise, and medical intervention for the reduction of subjective symptoms of dizziness in adult patients with cervicogenic dizziness: A systematic review and meta-analysis

Chiropractic and Manual Therapies / 2018;26(Supplement 1):

Netherlands BioMed Central Ltd. 2018 /

19/07/21 Poster/conference abstract

Sawicki, Carolyn P; Zimcik, Heather; McGillivray, Colleen

A Case of Dizziness After Traumatic Cervical Spinal Cord Injury.

American journal of physical medicine & rehabilitation / 2020;99(12):1191-1194

United States 2020 /

03/09/21 Wrong patient population (dizziness of vascular, central, vestibular or pharmacological causes)

Scherer, H.

Vertigo of cervical origin

Nervenheilkunde / 1997;16(2):93-97

Germany Schattauer GmbH (P.O. Box 104543, Stuttgart D-70174, Germany) 1997 /

22/07/21 published before 2000

Seifert, K.

Differential diagnosis and therapy of vertigo of cervical origin

Laryngo- Rhino- Otologie / 1990;69(7):394-397

Germany 1990 /

09/09/21 published before 2000

Seo, MW

Botulinum toxin treatment in cervical dizziness

EUROPEAN JOURNAL OF NEUROLOGY 2007;14():111-112

2007

07/09/21 Poster/conference abstract

Sun YZ, Zhang DY, Li Y, Cui FL

Clinical observations on acupuncture at heel vessels for the treatment of cervical vertigo

/ 2015;34(12):1214

2015 /

12/09/21 Poster/conference abstract

Suska, J

Manual Therapy for Cervicogenic Dizziness: Long-Term Outcomes of a Randomised Trial

PHYSIOSCIENCE 2016;12(2):80-+

2016

21/07/21 Other language then french, english, spanish, italian and Portuguese

Taura, Akiko

Cervical vertigo

Equilibrium Research / 2018;77(2):47-57

Japan Japan Society for Equilibrium Research 2018 /

19/07/21 Other language then french, english, spanish, italian and portuguese

Taura, Akiko; Shimizu, Kyoko; Hosomi, Yoshiko; Torii, Hiroko; Ogino, Eriko; Ito, Juichi; Ogita, Hideaki; Funabiki, Kazuo

Characteristic findings of stabilometry in patients with cervical vertigo

Equilibrium Research / 2012;71(2):87-95

Japan Japan Society for Equilibrium Research (39 Kawabata Higash Maruta-cho, Sakyo-ku, Kyoto 606, Japan) 2012 /

19/07/21 Other language then french, english, spanish, italian and Portuguese

Tavanai, Elham; Hajiabolhassan, Fahimeh

Cervicogenic vertigo: etiology, diagnosis and treatment.

Audiology 08// 2013;22(3):1-13

Tehran University of Medical Sciences 2013 08//

17/07/21 Other language then french, english, spanish, italian and portuguese

Taylor, A.; Kerry, R.

Structured debate as a tool for augmenting clinical reasoning in the cervical spine; Does dizziness equal danger?

Physiotherapy (United Kingdom) / 2011;97(SUPPL. 1):eS1610

Elsevier Ltd 2011 /

17/08/21 Poster/conference abstract

Treleaven, J.; Landen Ludvigsson, M.; Kammerlind, A.-S.; Peolsson, A.; Peterson, G.

Balance, dizziness and proprioception in chronic whiplash with dizziness: An RCT comparing three exercise programs

Physiotherapy (United Kingdom) / 2015;101(SUPPL. 1):eS1535

Elsevier Ltd 2015 /

17/08/21 Poster/conference abstract

von Heymann, W

Headache, dizziness, tinnitus and cervical spine

MANUELLE MEDIZIN 2015;53(5):361-371

2015

19/07/21 Other language then french, english, spanish, italian and Portuguese

Wang, Z.-X.; Chen, G.-H.; Zhang, J.-Y.; Shi, X.-C.; Wu, L.-C.; Feng, Z.-W.; Wang, K.; Ding, H.-C.

Clinical analysis of acupuncture combined with tuina in treating cervical vertigo

Journal of Acupuncture and Tuina Science 2014;12(5):306-309

2014

10/10/21 Wrong patient population (dizziness of vascular, central, vestibular or pharmacological causes)

WANGJ, QINX, ZHANGX

Clinical Observation on 35 Cases of Cervical Vertigo in the Acute Stage Treated by the Heated Medicinal Pillow and Betahistine

/ 2011;4():017

01/08/21 Other language then french, english, spanish, italian and Portuguese

Watanabe, Naohiko; Kikuchi, Hitoshi

Investigation of dizziness caused by head and neck injury

Equilibrium Research / 2005;64(6):472-478

Japan Japan Society for Equilibrium Research (39 Kawabata Higash Maruta-cho, Sakyo-ku, Kyoto 606, Japan) 2005 /

18/08/21 Other language then french, english, spanish, italian and portuguese

Wei X H, Wang B, Zhang F L, Zhang L, Qiang N P

The influence of herbal hot ironing, pressing beans on auricular point combined with syndrome differentiation and care on therapeutic effects of cervical vertigo

/ 2016;29(8):135

2016 /

12/09/21 Poster/conference abstract

Weikert, S.; Gabel, P.; Scholz, P.; Scherer, H.; Orawa, H.; Holzl, Matthias

Investigations concerning the head-trunk-coordination

Laryngo- Rhino- Otologie / 2008;87(4):245-251

Germany Georg Thieme Verlag (Rudigerstrasse 14, Stuttgart D-70469, Germany) 2008 /

21/07/21 Other language then french, english, spanish, italian and Portuguese

Wiest, G.

A neurological perspective on so-called cervical vertigo

Journal fur Neurologie, Neurochirurgie und Psychiatrie / 2016;17(1):7-12

Austria Krause und Pachernegg GmbH (Mozartgasse 10, Gablitz A-3003, Austria) 2016 /

10/09/21 Other language then french, english, spanish, italian and Portuguese

Wise, C.H.; Schenk, R.J.

Clinical decision making in the application of high-velocity, low-amplitude thrust manipulation of the cervical spine: A review of the literature

Journal of Manual and Manipulative Therapy / 2010;18(4):238-239

Maney Publishing 2010 /

19/07/21 Poster/conference abstract

Wrisley, D.M.; Sparto, P.J.; Whitney, S.L.; Furman, J.M.

Cervicogenic dizziness: A review of diagnosis and treament

Journal of Orthopaedic and Sports Physical Therapy / 2000;30(12):755-766

United States Movement Science Media 2000 /

10/10/21 duplicate

Wrisley DM

Off balance: cervicogenic dizziness can be difficult to diagnose and treat.

Advance for Directors in Rehabilitation Nov 2001;10(11):57-58

Pennsylvania Merion Publications 2001 Nov

12/09/21 expert advice/commentaries/letters.

Wu Ji-Sheng, Xv Que-Ying

Cervical vertigo treated with acupuncture and costus root : 50 cases reported

/ 2003;19(3):12 2003 /

12/09/21 Poster/conference abstract

Wu XP

Prospective study of three projects of treatment on chronic obstinate vertigo of cervical origin

/ 2002;6(14):2172

2002 /

12/09/21 Poster/conference abstract

Xiaoxiang, Zeng

Jinger moxibustion for treatment of cervical vertigo --a report of 40 cases.

Journal of traditional Chinese medicine = Chung i tsa chih ying wen pan / 2006;26(1):17-8

China 2006 /

02/08/21 Wrong patient population (dizziness of vascular, central, vestibular or pharmacological causes)

Xie Q, Wang Q

A Randomized controlled trial of manipulation combining with traction in the treatment of middle and old-aged patients with cervical vertigo

/ 2008;(11):

2008 /

09/09/21 Poster/conference abstract

Xue Xilin

Cervical plexus puncturing plus moxibustion for treatment of 78 cases of cervical vertigo

/ 2001;11(3):15

2001 /

09/10/21 Poster/conference abstract

Yamauchi, T.

Statistical analysis of elderly patients with vertigo or dizziness

Otolaryngology - Head and Neck Surgery (Japan) 2012;84(11):837-841

2012

05/10/21 Other language then french, english, spanish, italian and portuguese

Yang J, Wang F, Xiao WH

Evaluation on the rating scale of the treatment of patients with cervical vertigo with the method of acupuncture at Sishencong

/ 2014;23(12):2291

2014 /

09/09/21 Poster/conference abstract

Yan LiAng

Clinical observation of 30 cases of cervical vertigo treated with compound armillaria mellea preparation tablets and nimodipine

/ 2009;4(1):34 2009 /

01/08/21 Other language then french, english, spanish, italian and Portuguese

You L

Clinical observation of the effect of scalp acupuncture on symptoms and functions of patients with cervical vertigo

/ 2014;23(4):171

2014 /

12/09/21 Poster/conference abstract

Zeng, Xi; Chen, Shangkang; Guan, Chenxia; Peng, Yuzhong; Jiang, Li

Effects of mental intervention on cervical vertigo

Chinese Journal of Clinical Rehabilitation / 2003;7(1):160

China Journal of Clinical Rehabilitative (P.O. Box 1200, Shenyang 110004, China) 2003

/

12/09/21 Poster/conference abstract

Zhao XW, Bi FB

Treating cervical vertigo by rotatiing and tracting manipulation and herbal decoction

/ 2015;10(9):1391

2015 /

12/09/21 Poster/conference abstract

Zhou Lis-Shah

Observations on the curative effect of warmed-needle acupuncture of jiaji points as main ones on cervical vertigo

/ 2001;20(6):12

2001 /

12/09/21 Poster/conference abstract

Zhu, Xinwei; Han, Jianlong; Zang, Rui; Qiu, Siqiang; Chang, Gang; Zuo, Jinliang

Functional Pathway Between Cervical Spinal and Sympathetic Ganglia: A Neurochemical Foundation Between Neck Pain and Vertigo.

Pain physician / 2019;22(6):E627-E633

United States 2019 /

02/08/21 Articles on animals

Zielinska-Blizniewska, Hanna; Pietkiewicz, Marzena Bielinska Piotr; Milonski, Jaroslaw; Olszewski, Jurek

Evaluation of post-isometric relaxation influence on muscle tone in cervical vertigo patients

Aktualnosci Neurologiczne / 2012;12(3):169-175

Poland Medical Communications (Ul.Ojcowska 11, Warszawa 02-918, Poland)

19/07/21 Other language then french, english, spanish, italian and portuguese

Zielinska-Blizniewska, Hanna; Bielinska, Marzena; Pietkiewicz, Piotr; Olszewski, Jurek

Subjective and objective evaluation of the results of cervical vertigo kinesitherapy

Otolaryngologia Polska / 2011;65(3 SUPPL.):93-99

Poland Elsevier Urban and Partner sp. z o.o. (ul. Kosciuszki 29, Wroclaw 50-011, Poland) 2011 /

21/07/21 Other language then french, english, spanish, italian and Portuguese

NCT03291912

Chuna manual therapy for cervicogenic dizziness

Therapeutic effect of adjuvant chuna manual therapy (CMT) in subjects with cervicogenic dizziness: a prospective, pragmatic, assessor-blind, randomized controlled trial / 2017;():

2017 / research protocols

DRKS00022200

What are the effects of manual therapy on the musculature in potentially cervicogenic tinnitus and dizziness symptoms? A randomized study

/ 2020;(): 2020 /

16/07/21 Other language then french, english, spanish, italian and portuguese

NCT02772042

Effectiveness of Traction Manipulation of Upper Cervical Spine on Cervicogenic

Dizziness / 2016;():

2016 /

09/09/21 research protocols

NCT04347148

Postural and Clinical Outcomes of SNAGs Treatment in Cervicogenic Dizziness Patients:

a Randomised Controlled Trial / 2020;():

2020 /

09/09/21 research protocols

NCT03485768

PDCN for Treatment of Cervical Vertigo

Therapeutic Effects and Safety of Percutaneous Disc Decompression With Coblation

Nucleoplasty in Cervical Vertigo / 2018;():

2018 /

09/09/21 research protocols

ChiCTR-ION-16009815

A study on the optimization of kinetic parameters and the mechanism of the massage treatment of vertigo

/ 2016;(): 2016 /

12/09/21 research protocols

ChiCTR-INR-17012061

Clinical study on the treatment of cervical dizziness by intra-disc injection of methylene blue

/ 2017;(): 2017 /

12/09/21 research protocols

ChiCTR-IOR-16009919

Epidural block plus electro-acupuncture versus epidural block alone for cervical vertigo: a pragmatic randomized controlled trial

/ 2016;(): 2016 /

12/09/21 research protocols

ChiCTR-TRC-14005154

Cervical rotation-traction manipulation for cervical vertigo: a multicenter randomized controlled trial

/ 2014;(): 2014 /

12/09/21 research protocols

The clinical research on cervical vertigo treated with houxi & shenmai acupuncture

/ 2007;(): 2007 /

12/09/21 Poster/conference abstract

ChiCTR-ONRC-13003186

clinical study of cervical manipulation for cervicogenic dizziness

clinical study of cervical manipulation for cervicogenic vertigo/dizziness / 2013;():

2013 /

12/09/21 research protocols
